# Color and Near‐Infrared Reflectance Covary in Distinct Ways Across Taxa

**DOI:** 10.1002/ece3.73381

**Published:** 2026-04-19

**Authors:** Jonathan Goldenberg, Jessica L. Dobson, Gerben Debruyn, Michaël P. J. Nicolaï, Svana Rogalla, Bram Vanthournout, Federico Massetti, Susana Clusella‐Trullas, Diederik Strubbe, Katrien De Wolf, Dries Bonte, Bastiaan Star, Matthew D. Shawkey, Liliana D'Alba

**Affiliations:** ^1^ Department of Biology, Evolution and Optics of Nanostructures Group Ghent University Ghent Belgium; ^2^ Department of Biosciences, Center for Ecological and Evolutionary Synthesis University of Oslo Oslo Norway; ^3^ Department of Recent Vertebrates Royal Belgian Institute of Natural Sciences Brussels Belgium; ^4^ Instituto Biofisika, Basque Centre for Biophysics (CSIC‐UPV/EHU) University of the Basque Country (UPV/EHU) Leioa Spain; ^5^ University of Applied Sciences and Arts (HOGENT), Research Center AgroFoodNature Gent Belgium; ^6^ Department of Botany and Zoology Stellenbosch University Stellenbosch South Africa; ^7^ School for Climate Studies Stellenbosch University Stellenbosch South Africa; ^8^ Research Institute for Nature and Forest Brussel Belgium; ^9^ Department of Biology, Centre for Research on Ecology, Cognition and Behaviour of Birds (ECoBird) Ghent University Ghent Belgium; ^10^ Terrestrial Ecology Unit, Department of Biology Ghent University Ghent Belgium; ^11^ Naturalis Biodiversity Center Leiden the Netherlands

**Keywords:** color properties, coloration, thermoregulation, Vis–NIR relationship

## Abstract

Solar radiation reaching Earth spans 300–2500 nm, covering ultraviolet (UV), visible (Vis), and near‐infrared (NIR) wavelengths. Its reflectance is critical for animal signaling, camouflage, and thermoregulation, likely leading to selective trade‐offs that shape diverse reflectance patterns. Nonetheless, little is known about associations among these spectral regions across different taxa. Here, we investigate if the reflectance in different regions of the spectrum (UV, Vis, NIR) is correlated using data from integuments and eggs of 322 endotherms and ectotherms. To disentangle these relationships, we examined correlation strength, slope, and intercept, thereby providing nuance on consistency of association, magnitude of change across wavelengths, and baseline differences among taxa. We find that UV and Vis reflectance are significantly positively correlated across all taxa, whereas Vis–NIR correlation strengths are variable. For instance, in integuments, while the slopes of the Vis–NIR reflectance are positively correlated, their intercepts differ among taxa, suggesting that baseline NIR reflectance relative to Vis is influenced by macromolecular composition and/or structure. Moreover, in eggshells, UV–Vis and Vis–NIR slopes are also positively correlated, except for birds that show no significant Vis–NIR correlation. Our findings suggest that reflectance across wavelengths can be decoupled, laying a foundation for understanding how reflectance traits evolve under physiological and ecological pressures.

## Graphical abstract

Solar reflectance across ultraviolet, visible, and near‐infrared wavelengths was analyzed in integuments and eggs of 322 species. We found consistent positive correlations between UV and visible reflectance, while visible‐NIR associations varied across taxa, with differences in baseline reflectance suggesting structural or compositional influences. These results show that spectral regions can be decoupled, highlighting evolutionary trade‐offs in reflectance traits.

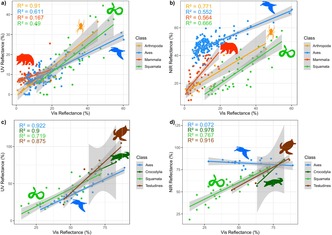

## 1 Introduction

Animals' integuments reflect sunlight across the ultraviolet (UV), visible (Vis), and near‐infrared (NIR) regions, which strongly influences visual signaling, camouflage, and thermoregulatory functions (Norris and Lowe 1964; Endler et al. 2005; Smith et al. 2016; Allen et al. 2020; Nicolaï, van Hecke, et al. 2024; Nicolaï, Rogalla, et al. 2024). This reflectance is determined by a combination of pigmentary and structural properties of the material (Shawkey and D'Alba 2017; D'Alba and Shawkey 2019). While UV–Vis reflectance has been widely studied in behavioral and ecological contexts (Shimizu et al. 2009; Ödeen and Håstad 2013; Crowell et al. 2024), research on NIR wavelengths has been more limited (Medina et al. 2018). While all portions of UV–Vis–NIR contribute to thermoregulation, UV–Vis also contributes to other functions such as visual signaling. As such, being released from other selective pressures, NIR is more closely tied to thermoregulation and structural functions, particularly in modulating heat absorption and dissipation (Shawkey et al. 2017; Wang et al. 2021). These functional differences suggest that selection pressures could drive divergence between the two ranges (Stuart‐Fox et al. 2017). For example, low UV–Vis reflectance may enhance camouflage but, if coupled with low NIR reflectance, could also increase heat gain, a trade‐off that may be advantageous in cool environments but detrimental in warmer ones, potentially driving a decoupling of the two traits (Gómez et al. 2016; Smith et al. 2016; Medina et al. 2018).

The mechanisms by which organisms produce color vary greatly across taxa, contributing to differences in reflectance patterns. For instance, birds and mammals rely on pigmentary coloration, such as melanin and carotenoids, combined with structural elements such as nanostructures in feathers or fur that enhance or modify reflectance properties (Caro 2005; Eliason and Shawkey 2010). Bird plumage, in particular, exhibits complex structural coloration, including multilayered arrangements in feathers (Shawkey et al. 2009) that can influence UV–Vis and even NIR reflectance (Shawkey et al. 2017). Although ectotherms such as squamates also use mixes of pigment and structural‐based colors, they differ in the composition of those elements (e.g., use of pteridines as pigments or guanine crystals as structural elements) in their integuments, which cause distinct reflectance patterns (Vitt and Caldwell 2014; Teyssier et al. 2015; Nicolaï et al. 2021; Goldenberg et al. 2022). These different components of color production not only create diverse visual appearances but may also underlie differences in their thermal management strategies (Rogalla, Patil, et al. 2021). Similarly, arthropod coloration can result from both pigments and intricate structural arrangements (Hsiung et al. 2017; Hsiung et al. 2019; Badejo et al. 2020; Haque et al. 2024) such as chitin layers, which could influence their reflectance properties in thermally significant ways (Clusella‐Trullas and Nielsen 2020; Vanthournout et al. 2021; Ospina‐Rozo et al. 2023).

Biological differences among taxa, such as physiological mechanisms for thermoregulation and metabolic demands, also play a role in shaping reflectance properties (Walsberg and Wolf 1995; Azócar et al. 2016). Birds and mammals, as endotherms, rely on metabolic processes to maintain homeostasis, which could influence their NIR reflectance properties to balance heat retention and dissipation while managing the energetic costs associated with thermoregulation. For instance, Heppner (1970) showed that black plumage in birds can be associated with lower metabolic rates, likely due to its reduction of heat loss and energy expenditure. Similarly, mammals may exhibit specific adaptations in fur or skin pigmentation for thermal regulation (Caro 2005). By contrast, ectothermic taxa like squamates rely heavily on external pathways of heat exchange and may exhibit distinct reflectance patterns due to their thermoregulatory strategies (Goldenberg et al. 2021; Mader et al. 2022). These differences suggest that the relationship between UV–Vis and NIR reflectance could vary across groups, reflecting both their unique color‐producing mechanisms, evolutionary pressures, and physiological constraints. However, empirical tests of these patterns remain scarce, especially in a comparative framework across major vertebrate and invertebrate groups (Stuart‐Fox et al. 2017).

In addition to body integuments, similar reflectance trade‐offs may occur in other biologically relevant materials, such as eggshells. Unlike body tissues such as feathers or furs, eggshells are bioceramics that function as primary mediators between the environment and the embryo, and whose properties have been shaped in response to the challenges imposed by the environment at the incubation site (Guerra‐Grenier 2019; D'Alba et al. 2021; Debruyn et al. 2024). For instance, Bakken et al. (1978) showed that varying concentrations of protoporphyrin pigments in the shell explain NIR reflectance and thermal properties in bird eggs, suggesting that eggshell coloration is finely tuned to specific ecological pressures. But these patterns have only been studied in a limited number of bird species, and whether they are consistent across taxa remains unclear. Furthermore, color‐producing mechanisms in eggs seem to be more limited, restricting the diversity of colors and optical properties of eggs (Gosler et al. 2005). Because eggshells tend to serve similar functions across species and are limited by color‐production constraints (Cassey et al. 2010; Hanley et al. 2015), their reflectance relationships may be more correlated. In contrast, body tissues are shaped by a wider range of physiological and behavioral factors (Dalrymple et al. 2018; Babarović et al. 2023), leading to potentially greater variability in cross‐range reflectance patterns.

To investigate such correlations, we compiled and analyzed spectrum‐wide reflectance data from 332 species representing mammals, birds, squamates, crocodiles, testudines, and arthropods. Reflectance measurements were taken from feathers, fur, and scales (hereafter referred to as “integument”), and eggshells to test whether UV and Vis reflectance co‐vary with NIR across these taxa and whether these relationships differ between groups or biomaterial types. We hypothesized that the relationship between UV–Vis and NIR reflectance should vary among groups due to differences in metabolic rates, thermoregulatory strategies, and structural adaptations. To capture these differences, we considered multiple aspects of the relationship: correlation strength (*r*) to assess consistency of association, slope to evaluate the magnitude of change across wavelengths, intercept to identify baseline differences in reflectance, and *R* ^2^ to quantify the proportion of variance explained by predictors. Specifically, we expected endotherms (birds and mammals) to exhibit relatively consistent positive correlations between NIR and UV–Vis reflectance—reflected in higher *r* values and comparable slopes—driven not only by thermoregulatory considerations, particularly during active periods or in hot climates, but also by the underlying mechanism of color production: integumentary structures and pigments that generate visible coloration often simultaneously influence NIR reflectance, leading to coordinated spectral patterns (Shawkey et al. 2017). Thus, although a stable internal body temperature through metabolic heat production may buffer the direct selective pressure on reflectance traits, structural coupling of Vis and NIR remains likely. For ectotherms (squamates and our sampled arthropods) we predicted that they would show weaker or more variable correlations between NIR and UV–Vis reflectance than endotherms, reflected in lower correlation strength (*r*). Because ectotherms rely on external heat sources and behavioral strategies such as basking or shade‐seeking for thermoregulation, selection may favor more flexible reflectance properties; this flexibility may reduce the extent of coordinated spectral reflectance across wavelengths, leading to functional decoupling of visible and NIR traits. As such, differences in how taxa prioritize visual appearance versus heat gain or minimizing overheating likely shape their reflectance patterns. Moreover, we predicted that eggshells, which have reflectance properties that are constrained by shared functional requirements (Wisocki et al. 2019; Boisseau and Woods 2024), should show higher consistency of reflectance correlations between NIR and Vis across taxa. We emphasize that our study does not address visual communication or perception (e.g., whether animals detect particular wavelengths); instead, we focus on correlations among spectral regions of reflectance. We acknowledge, however, that reflectance patterns may be influenced by multiple selective pressures including thermoregulation, camouflage, signaling, or other ecological functions and environmental parameters (Goldenberg et al. 2024). Here, we extend previous work highlighting the roles of pigmentation, structure, and thermoregulation in shaping reflectance (Stuart‐Fox et al. 2017) by examining how these relationships scale across a broader phylogenetic and integument‐based frameworks, providing new insights into the evolutionary and possible functional constraints underlying reflectance variation.

## 2 Material and Methods

### 2.1 Species Selection and Reflectance Data

We compiled reflectance data across the UV (350–380 nm), Vis (380–740 nm), and NIR (740–2000 nm) spectra for a total of 332 species (Figure S1) from a combination of previously published studies (Goldenberg et al. 2021, 2023; Rogalla, Nicolaï, et al. 2021; Mader et al. 2022) and unpublished data collected over the past decade by the Evolution and Optics of Nanostructures (EON; Ghent University) and Climate and Invasions: Mechanisms in Ectotherms (CLIME; Stellenbosch University) groups. As such, our sampling reflects the availability of long‐term datasets rather than a priori targeting of specific geographic regions or taxonomic groups. Although the UV, Vis, and NIR regions differ substantially in spectral width, they represent biologically distinct regions, with different properties, of solar radiation that are ecologically and physiologically relevant. The UV interval (350–380 nm) is narrower than the Vis and NIR ranges because this window corresponds to the portion of the UV spectrum where integumentary materials such as keratin, melanin, and chitin exhibit measurable reflectance; below ~350 nm, reflectance is typically negligible or unreliable due to strong absorption and instrument noise, whereas wavelengths above 380 nm fall within the visible range for most vertebrates. Because our study focuses on thermal rather than visual hypotheses, this UV window captures the biologically relevant portion of short‐wavelength reflectance for assessing cross‐range thermal relationships. Our statistical approach reflects these functional divisions, rather than assuming equal spectral intervals. Acquisition of new data followed standardized protocols established by our research groups to ensure comparability across datasets. Specifically, all reflectance data has been measured across the 350–2500 nm range and collected either through (1) a dual spectrophotometer (Avantes Inc., Broomfield, CO, USA) and light source (AvaLight‐DH‐S Deuterium‐Halogen Light Source and AvaLight‐HAL‐(S)‐MINITungsten Light Source) setup connected to a quadrifurcated fiber optic cable and held at 90° from the sampled surface using a RPH‐1 probe holder (317 species) or via (2) a field spectrophotometer (FieldSpec 3, ASD Inc., Colorado, USA) coupled with a ASD Fiber Optic Illuminator connected to a bifurcated probe with 6‐around‐1 configuration on the distal end, and held at 90° (15 species). Because our analyses focused on the relationships between spectral ranges (e.g., Vis–NIR) taken at the individual level using the same methods, rather than absolute reflectance values, differences in equipment specifications should not affect the results. All samples were either acquired from living or museum specimens. In no case did we measure alcohol‐preserved specimens as this preservation impacts the color properties of animal integuments. Moreover, for museum specimens we took precautionary measures and excluded individuals that showed physical damage and dusting. Our analysis focused on mean brightness (hereafter reflectance), using the *B2* parameter—see the formula below—from the “pavo2” (Maia et al. 2019) R package, as this metric is directly tied to absorption properties (Shawkey et al. 2017).

$$B_{2}=\frac{\sum_{\lambda_{\min}}^{\lambda_{\max}}R_{i}}{n_{w}}=\frac{B_{1}}{n_{w}}$$

where:

- *λ*_*max*_ and *λ*_*min*_ are the upper and lower wavelength limits for each spectral range (UV, Vis, NIR).
- *R*_*i*_ is the reflectance at wavelength *λ*_*i*_,
- *B*_*1*_ is the total brightness,
- *n*_*w*_ is the number of wavelengths.

Because *B2* represents mean reflectance across each spectral interval, it is not sensitive to differences in bandwidth, ensuring that the narrower UV range does not mechanically inflate reflectance values or cross‐range correlations. The dataset was categorized into two main material types, reflecting broad material property differences: integument and eggshells. For integumentary reflectance, we analyzed 275 adult—to account for possible ontogenetic color shifts—species across four major taxonomic groups: birds (*n* = 169), mammals (*n* = 50), squamates (*n* = 44), and arthropods (*n* = 12). For comparative purposes, repeated reflectance measurements for each species were averaged across dorsal body regions, defined as areas directly exposed to sunlight, to provide a representative dataset capturing ecologically relevant reflectance properties. Although sampling reflects the availability of long‐term datasets rather than systematic taxon‐level targeting, the species included span the major pigmentary and structural coloration mechanisms, ecological contexts, and material types characteristic of each group. This breadth provides a representative foundation for macro‐comparative analyses of UV–Vis–NIR reflectance.

For each exposed body region and individual, we averaged all reflectance (*B2*) measurements taken within that region to obtain a single mean value per individual, and then calculated species‐level means from these individual averages. To account for species with patterned coloration (i.e., with multiple color patches), such as the viper *Daboia palaestinae* , reflectance was measured across all major color regions present in the exposed areas. While intraspecific variation may exist, our focus on broad‐scale comparisons ensures that differences across taxa are biologically meaningful rather than artifacts of individual variability. Specifically for birds, where exposed body regions can show substantial intraspecific color variation and many areas receive direct light (Hernández‐Palma 2016), we restricted measurements to dorsal plumage, as this region is generally less affected by sexual selection and seasonal molt than head, breast, or wing patches (Hernández‐Palma 2016), making it more appropriate for broad comparative analyses. Moreover, prior work shows that cross‐range reflectance correlations are strong and consistent in birds despite intraspecific variation (e.g., Shawkey et al. 2017; Stuart‐Fox et al. 2018). For one spider species, *Araneus diadematus* , we included unpublished data from 31 individuals, ensuring that measurements were taken only from the background abdominal color to avoid the bright reflective patches that exhibit extremely high reflectance. To assess potential sampling effects, we specifically evaluated the relationship between Vis and NIR reflectance within this species. Despite substantial individual variation in absolute reflectance, the correlation between NIR and UV–Vis reflectance was strong (*R* = 0.9; Figure S2), justifying the use of average values for this species to analyze and interpret macroscale patterns. Due to the limited representation of arthropods in our dataset, results for this group are presented solely for visualization and to encourage further research on this taxon. For eggshell reflectance, three measurements along the equatorial region were obtained from 64 species, covering a diverse representation of taxa: birds (*n* = 20), squamates (*n* = 30), testudines (*n* = 11), and crocodiles (*n* = 3). As with arthropod integuments, results for testudine and crocodile eggshells are presented solely for visualization purposes.

Our sampling effort aimed to include both endothermic and ectothermic species, facilitating analyses of potential decoupling of reflectance properties across spectral regions. We provide in our repository the full species‐level source list (which links to the associated methods), whether specimens have been collected from museum or living individuals, and the spectrophotometer used.

### 2.2 Phylogenetic Tree Construction and Signal

For each taxonomic group we used the most recent available time‐calibrated, gene based phylogenetic trees (Zheng and Wiens 2016; Álvarez‐Carretero et al. 2022; Kumar et al. 2022; McTavish et al. 2024). To construct a comprehensive supertree, we combined these individual trees using the *tree.bind* function in “mulTree” (Healy et al. 2014). We estimated divergence times among taxa based on the median diversification times provided by the TimeTree of Life Project (http://www.timetree.org; Kumar et al. 2022). For species lacking genetic data, we interpolated their placement in the tree using available genetic information from congeneric species, ensuring broader phylogenetic coverage (see our repository for a detailed overview of taxonomic assignments). Next, to examine phylogenetic signals in integument and eggshell reflectance traits, we used *phylosig* in “phytools” (Revell 2024) to compute Pagel's Lambda (*λ*) coefficients.

### 2.3 Statistical Analyses

As there were strong phylogenetic signals (see results), we employed phylogenetic generalized least squares (PGLS) models implemented in “caper” (Orme 2023) to test for associations between reflectance ranges across species. Since reflectance data were expressed as continuous percentages, we did not transform our data before analyses. Specifically, for each material type, we constructed three models corresponding to responses in the UV, Vis, or NIR reflectance ranges. In each model, mean reflectance from one range served as the dependent variable, while the other two were independent variables. Because *B2* reflects band‐averaged absorptance, relationships among spectral regions are broad and monotonic rather than nonlinear, supporting the use of linear PGLS models for interpretability. We found very strong collinearity between the UV and Vis spectra (*r* integument‐eggs: 0.76–0.86, *p* < 0.001). While this may partly reflect the narrow bandwidth of the UV range (30 nm) and the continuous nature of reflectance spectra, we retained UV and Vis as separate predictors because they represent biologically and evolutionarily distinct regions of light. UV reflectance often arises from different structural or pigmentary mechanisms than those producing Vis coloration (Shawkey et al. 2017), and it can be subject to distinct selective pressures, such as thermoregulation and UV protection (Wisocki et al. 2019). Thus, despite the potential for statistical coupling, we consider it biologically meaningful to evaluate UV and Vis reflectance independently. As such, to avoid correlation between predictors, we ultimately ran two separate models, each with Vis as the independent variable: one using UV as the dependent variable, and the other using NIR. We also included taxonomic group as a fixed effect to test whether relationships among UV, Vis, and NIR reflectance varied across taxa. For taxa with significant effects, we conducted taxon‐specific PGLS analyses. From each model, we extracted slope, intercept, Pearson correlation coefficient (*R*), and coefficient of determination (*R* ^2^) values to assess differences in magnitude (slope), baseline (intercept), correlation strength (*R*), and the proportion of variance explained by the predictor(s) (*R* ^2^) across taxa.

We performed all analyses and visualizations in R v.4.1.3 (R Core Team 2025).

## 3 Results

We found strong phylogenetic signals across the entire dataset for all reflectance ranges for integument (*λ* = 0.95–0.97; *p* < 0.001) and eggshell (*λ* = 0.48–0.72; *p* < 0.001) materials.

**FIGURE 1:**
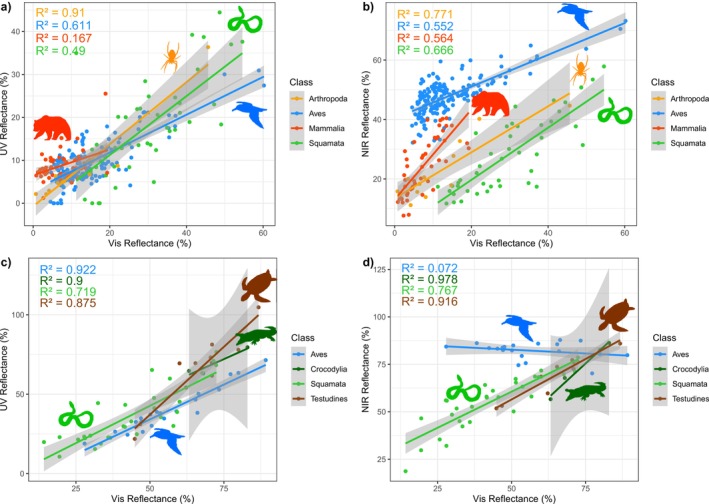
Relationship between UV, Vis, and NIR spectra across taxa. Predicted relationship between different reflectance ranges for integument (a, b) and eggshell (c, d) materials. Dots represent reflectance data from our input dataset. Slopes are estimates of pgls models and shaded areas show the confidence intervals. Output produced in “ggplot” (Wickham 2016) and pgls in “caper” (Orme 2023). Note that results for arthropods, crocodiles, and testudines are shown for visualization purposes only due to limited sample representation. For (a, b), number of species (*n*) Arthropoda = 12, *n* Aves = 169, *n* Mammalia = 50, *n* Squamata = 44; for (c, d), *n* Aves = 23, *n* Crocodylia = 3, *n* Squamata = 45, *n* Testudines = 7. Silhouette images from phylopic.org; *Araneus diadematus* by Birgit Lang under Attribution 3.0; all other silhouettes under CC0 1.0 Universal Public Domain Dedication.

We then evaluated how different reflectance regions are related to each other. Vis reflectance is significantly positively correlated with UV reflectance across all taxa and material types (*p* < 0.001, *R* ^2^ 0.17–0.92; Figure 1a,c; Tables S1 and S2). By contrast, the relationship between Vis and NIR reflectance shows more variation. For integumentary samples, Vis reflectance is a significant positive predictor of NIR across all taxa (*p* = 0.001); however, birds exhibit a significantly higher NIR reflectance than the other groups (*p* < 0.001; Figure 1b; Tables S3 and S4). In mammals (slope *m* = 1.51), NIR increases more steeply with Vis reflectance than in other groups (Figure 1b and Tables S5–S7). This difference in slope was statistically supported, with the Vis × Class interaction of mammals showing a steeper slope than both birds (interaction estimate = 0.859, *p* < *0.001*) and squamates (interaction estimate = 0.276, *p* = 0.011). Moreover, intercepts also differ across taxa, indicating baseline differences in NIR reflectance relative to Vis (Figure 1a,b). Fisher's *Z*‐transformed post hoc comparisons of group‐specific *R* values reveal no statistically significant differences in correlation strength (Table S8).

Although eggshell sample sizes were relatively small, we observed notable slope differences in the Vis–NIR relationship among squamates, crocodiles, and testudines compared to birds (Figure 1d; Tables S9 and S10), with squamates showing a positive slope (*m* = 0.84) and birds showing no significant relationship (*m* = −0.08). Post hoc comparisons from the PGLS model reveal a statistically significant difference in slope between squamates and birds (*p* < 0.001; Table S10). In parallel, Fisher's *Z* tests show a significant difference in correlation strength (*R*) between these two groups (*p* = 0.027; Figure 1d and Table S11).

## 4 Discussion

Our analyses revealed nuanced patterns of variance and co‐variance in UV, Vis, and NIR reflectance across taxa and material types, suggesting distinct mechanistic, evolutionary, ecological, and functional pressures. As predicted, UV–Vis reflectance exhibited similar patterns across taxa, while Vis–NIR relationships varied, hinting at an interplay between shared functional requirements and lineage‐specific responses. Variation occurred in both slopes, describing the rate of NIR change relative to Vis, and intercepts, suggesting baseline NIR differences that may reflect underlying macromolecular or structural properties like pigment composition or surface topography. In line with our hypotheses, ectothermic squamates showed a shallower Vis–NIR slope than mammals (Figure 1b). However, while slope magnitude was lower, the relationship remained tightly coupled, contrary to our expectations of decoupling of visible color from NIR. Unexpectedly for endotherms, birds had a smaller Vis–NIR slope and elevated baseline NIR reflectance, suggesting pigment use and eggshell or integument structure can override predicted tight spectral coupling. Together, slope and intercept variation reveal how tightly spectral regions are linked across taxa, offering insight into whether reflectance properties are shaped by consistent mechanistic constraints or more variable evolutionary influences.

Specifically, Vis reflectance significantly predicted NIR reflectance of the integument, but the patterns of this association varied across taxa (Figure 1). Birds exhibited higher NIR reflectance relative to any other group. As homeotherms (Delhey et al. 2021; Terrill and Shultz 2023), birds maintain an elevated and constant body temperature and need to prevent excessive heat gain from the environment, especially in direct sunlight (Rogalla et al. 2022). Therefore, their reflectance properties, including a consistently higher NIR reflectance (Figure 1B), may help them regulate their internal temperature more efficiently by reflecting more NIR (Delhey et al. 2021). Although high NIR reflectance could, in principle, reduce passive heat gain in cold conditions, several factors mitigate this potential cost: many birds undergo seasonal changes in plumage density, structure, or coloration that enhance insulation or solar absorption in winter (Rogalla et al. 2022), and metabolic heat production and behavioral thermoregulation typically dominate heat balance under cold conditions (Ritchison 2023). As a result, elevated NIR reflectance is unlikely to impose substantial winter disadvantages, whereas avoiding overheating in high‐irradiance environments represents a more consistent selective pressure. Unlike mammals, the unique branching and 3D complexity of feathers likely enhance NIR reflectance by scattering and reflecting more light than mammalian hair or the smooth surfaces of scales and cuticles in other taxa (Saranathan et al. 2012; Medina et al. 2018; Stuart‐Fox et al. 2018). Feathers also contain diverse pigment classes including carotenoids, melanins, and structural coloration mechanisms (Hill and McGraw 2006) which could influence how different wavelengths interact with avian integuments. These factors may contribute to the smaller slope between Vis and NIR reflectance observed in birds. Given that birds use visible coloration extensively for sexual signaling and ecological interactions (Hill and McGraw 2006), their potential ability to modulate NIR reflectance independently from visible wavelengths may be mechanistically linked to their pigment diversity and feather microstructure rather than solely an adaptive response to thermoregulation. Nevertheless, this structural complexity could provide birds with a mechanism to manage heat gain from NIR separately from the visible wavelengths that they use extensively for sexual signaling and other functions.

Mammals, on the other hand, showed a steeper increase in NIR reflectance with Vis reflectance than birds and squamates. While this trend aligns with their thermoregulatory needs as endotherms (Buckley et al. 2012), it is also likely influenced by their uniform use of melanin pigments in their integument (Hearing and Tsukamoto 1991). Melanin absorbs a broad spectrum of wavelengths, including NIR (Xie et al. 2024), potentially helping to explain why Vis and NIR are correlated in mammals. Unlike birds or squamates, mammals lack carotenoid pigments (with few exceptions (Galván et al. 2016)), and other reflective structural modifications that might otherwise increase NIR reflectance (Galván et al. 2016). Consequently, their integumentary reflectance properties may be influenced by both the constraints of their largely melanin‐based coloration mechanism, as well as selective pressures. These mechanistic limitations primarily influence baseline NIR reflectance, whereas the rate at which NIR increases with Vis reflectance reflects broader thermoregulatory and lineage‐specific patterns. Additionally, hair's simple structure and arrangement may inherently limit reflectance efficiency, reducing NIR scattering compared to feathers or hard‐bodied taxa like arthropods.

By contrast, squamates showed a shallower Vis–NIR slope. Although their color‐producing mechanisms may play a role in this relationship, these ectothermic organisms rely on external heat sources to regulate their body temperature (Goldenberg et al. 2022), meaning that in addition to heat absorption mediated by skin reflectance, heat exchange also takes place via other pathways, such as conduction and convection. Nonetheless, coloration may still contribute to the overall thermal balance of ectotherms (Clusella Trullas et al. 2007). For example, darker coloration is often assumed to enhance heat absorption in cooler environments, while lighter hues reduce heat gain in hotter climates (Goldenberg et al. 2025). Our findings indicate that the positive correlations between UV–Vis and NIR reflectance are consistent across all taxa but vary in magnitude, suggesting that, while darker colors generally correspond to higher NIR absorption, the degree of this effect differs among taxa. This variation in magnitude could reflect differences in how taxa integrate reflectance with their thermal strategies: birds combine high NIR reflectance with insulation and structural complexity, mammals show strong Vis–NIR coupling shaped by melanin‐based pigmentation, and squamates rely more heavily on behavioral thermoregulation, resulting in shallower slopes. This difference between endothermic and ectothermic organisms underscores the distinct thermoregulatory pressures that shape reflectance patterns (Goldenberg et al. 2022).

Similarly, eggshells, which primarily function in embryonic protection (D'Alba and Shawkey 2015), camouflage (Lovell et al. 2013), and thermal regulation (Grant 1982), have variable Vis–NIR relationships. The slope differed between squamates and birds, suggesting that even within eggshells, reflectance properties are taxon‐specific. Unlike the integument data, this variation might be more structurally linked, as protoporphyrin pigments and calcite (i.e., the main components of avian eggshells), exhibit high NIR reflectance (> 90%), enhancing thermal reflection without compromising visibility (Bakken et al. 1978). In contrast, reptilian eggshells lack strong pigmentation and are typically pale or white, with reflectance driven by the physical properties of the calcareous shell matrix. This structural uniformity likely underlies the stronger Vis–NIR correlation observed in squamates than birds. Ecological conditions at the nesting or incubation site may further modulate these patterns, as exposure to solar radiation, substrate type, and microclimate can impose different thermal and camouflage demands on eggs across taxa (D'Alba et al. 2021). As such, reflectance properties in squamate eggshells may arise largely from underlying material composition and structural uniformity, with ecological pressures acting on a more constrained palette of optical traits.

While this study offers valuable insights, limitations such as small sample sizes, broad taxonomic categories, and uneven sampling warrant consideration. Our hypothesis‐driven approach draws on the best available data, acknowledging constraints from non‐systematic sampling. Nevertheless, the dataset spans major pigmentary and structural coloration mechanisms, ecological contexts, and material types typical of each taxa, allowing to capture broad comparative patterns. Recurring patterns across taxa support the validity of our findings and highlight the need for expanded sampling, particularly among underrepresented groups. Because our analyses rely on band‐averaged reflectance (*B2*), they necessarily collapse fine‐scale spectral structure such as peaks, pigment‐specific absorption features, and structural resonances. As a result, the covariation we report reflects broad differences in overall albedo and absorptance across spectral regions rather than mechanistic coupling at specific wavelengths. Our inferences therefore pertain to thermal energy balance at the band level. While complementary analyses incorporating spectral shape (e.g., PCA on full spectra, spectral slopes, or peak‐based descriptors) could provide additional mechanistic insight, such analyses are beyond the scope of our thermal‐focused comparative framework.

This work highlights the diverse ecological influences and suggests that underlying functional and mechanistic constraints likely shape reflectance traits across taxa and material types. The consistent UV–Vis relationships across taxa point to shared mechanisms, while the variable Vis–NIR patterns—noted previously by Stuart‐Fox et al. (2017)–reveals taxon‐specific responses likely reflecting distinct thermoregulatory and ecological demands. By emphasizing mechanistic considerations alongside evolutionary and ecological contexts, this work emphasizes the importance of an integrative approach to understanding reflectance variation across species.

## Author Contributions

**Jonathan Goldenberg:** conceptualization (equal), data curation (lead), formal analysis (lead), funding acquisition (supporting), investigation (lead), methodology (lead), visualization (lead), writing – original draft (lead), writing – review and editing (lead). **Jessica L. Dobson:** data curation (supporting), investigation (supporting), writing – original draft (supporting), writing – review and editing (supporting). **Gerben Debruyn:** data curation (supporting), investigation (supporting), writing – original draft (supporting), writing – review and editing (supporting). **Michaël P. J. Nicolaï:** data curation (supporting), investigation (supporting), writing – original draft (supporting), writing – review and editing (supporting). **Svana Rogalla:** data curation (supporting), writing – original draft (supporting). **Bram Vanthournout:** data curation (supporting), supervision (supporting), writing – original draft (supporting). **Federico Massetti:** data curation (supporting), writing – original draft (supporting). **Susana Clusella‐Trullas:** funding acquisition (supporting), supervision (equal), writing – original draft (supporting), writing – review and editing (supporting). **Diederik Strubbe:** investigation (supporting), writing – original draft (supporting). **Katrien De Wolf:** investigation (supporting), writing – original draft (supporting), writing – review and editing (supporting). **Dries Bonte:** funding acquisition (supporting), supervision (supporting), writing – original draft (supporting). **Bastiaan Star:** writing – original draft (supporting), writing – review and editing (supporting). **Matthew D. Shawkey:** conceptualization (supporting), funding acquisition (equal), supervision (equal), writing – original draft (supporting), writing – review and editing (supporting). **Liliana D'Alba:** conceptualization (lead), data curation (supporting), funding acquisition (equal), supervision (equal), writing – original draft (supporting), writing – review and editing (supporting).

## Funding

J.G. was supported by the Wenner‐Gren Foundation UPD2022‐0061 and funded by the European Union's Horizon Europe research and innovation program under the Marie Skłodowska‐Curie grant agreement No. 101126636. D.S. was supported by Methusalem Project 01M00221 (Ghent University) awarded to Frederick Verbruggen, Luc Lens and An Martel. B.V., D.B. and K.D.W. received funding from the Flemish Research Funds FWO G080221N. We further acknowledge funding from FWO G0E8322N, Human Frontiers Science Program RGP0047, Air Force Office of Scientific Research FA9550‐18‐1‐0477, FA9550‐23‐1‐0622, FA8655‐23‐2‐7041, and FWO/South Africa NRF research cooperation grant FLGR160625174165.

## Conflicts of Interest

The authors declare no conflicts of interest.

## Supporting information


**Figure S1:** Phylogenetic relationship across the 332 examined species.
**Figure S2:** Variation in the relationship between Vis and NIR in individuals of the cross spider, *Araneus diadematus* , showing a strong positive correlation between the two spectral regions.
**Table S1:** Output from the phylogenetic regression model testing the relationship between UV and Vis spectra in integuments across the four examined animal groups. “ClassArthropoda” and “Vis:classArthropoda” are the two reference levels.
**Table S2:** Output from the phylogenetic regression model testing the relationship between UV and Vis spectra in eggshells across the four examined animal groups. “ClassAves” and “Vis:classAves” are the two reference levels.
**Table S3:** Output from the phylogenetic regression model testing the relationship between NIR and Vis spectra in integuments across the examined animal groups. “ClassArthropoda” and “Vis:classArthropoda” are the two reference levels.
**Table S4:** Output from the posthoc test on the phylogenetic regression model testing the relationship between NIR and Vis spectra in integuments across the examined animal groups.
**Table S5:** Output from the phylogenetic regression model testing the relationship between NIR and Vis spectra in Aves.
**Table S6:** Output from the phylogenetic regression model testing the relationship between Vis and NIR spectra in Mammalia.
**Table S7:** Output from the phylogenetic regression model testing the relationship between Vis and NIR spectra in Squamata.
**Table S8:** Results from pairwise comparisons using Fisher's *Z*‐test to assess statistical differences in correlation strength (*R*) across taxa in the phylogenetic regression model testing variability in NIR–Vis spectra in integuments (Table S3). Upper table shows the *R* values and the bottom one the pairwise comparisons.
**Table S9:** Output from the phylogenetic regression model testing the relationship between NIR and Vis spectra in eggshells across the four examined animal groups. “ClassAves” and “Vis:classAves” are the two reference levels.
**Table S10:** Output from the posthoc test on the phylogenetic regression model testing the relationship between Vis and NIR spectra in eggshells across the four examined animal groups.
**Table S11:** Results from pairwise comparisons using Fisher's *Z*‐test to assess statistical differences in correlation strength (*R*) across taxa in the phylogenetic regression model testing variability in NIR–Vis spectra in eggs (Table S9). Upper table shows the *R* values and the bottom one the pairwise comparisons.

## Data Availability

All R‐scripts and data are deposited at https://doi.org/10.5281/zenodo.19210896.
