# Supplementary material for: Color and Near‐Infrared Reflectance Covary in Distinct Ways Across Taxa
Source: Ecol Evol. 2026 Apr 19;16(4):e73381. doi: 10.1002/ece3.73381 (PMC13092372; doi:10.1002/ece3.73381)
Supplement: Supplementary file 1 — Figure S1: Phylogenetic relationship across the 332 examined species. Figure S2: Variation in the relationship between Vis and NIR in individuals of the cross spider, Araneus diadematus , showing a strong positive correlation between the two spectral regions. Table S1: Output from the phylogenetic regression model testing the relationship between UV and Vis spectra in integuments across the four examined animal groups. “ClassArthropoda” and “Vis:classArthropoda” are the two reference levels. Table S2: Output from the phylogenetic regression model testing the relationship between UV and Vis spectra in eggshells across the four examined animal groups. “ClassAves” and “Vis:classAves” are the two reference levels. Table S3: Output from the phylogenetic regression model testing the relationship between NIR and Vis spectra in integuments across the examined animal groups. “ClassArthropoda” and “Vis:classArthropoda” are the two reference levels. Table S4: Output from the posthoc test on the phylogenetic regression model testing the relationship between NIR and Vis spectra in integuments across the examined animal groups. Table S5: Output from the phylogenetic regression model testing the relationship between NIR and Vis spectra in Aves. Table S6: Output from the phylogenetic regression model testing the relationship between Vis and NIR spectra in Mammalia. Table S7: Output from the phylogenetic regression model testing the relationship between Vis and NIR spectra in Squamata. Table S8: Results from pairwise comparisons using Fisher's Z‐test to assess statistical differences in correlation strength (R) across taxa in the phylogenetic regression model testing variability in NIR–Vis spectra in integuments (Table S3). Upper table shows the R values and the bottom one the pairwise comparisons. Table S9: Output from the phylogenetic regression model testing the relationship between NIR and Vis spectra in eggshells across the four examined animal groups. “ClassAves” and “Vis:clas [file ECE3-16-e73381-s001.pdf]

Supplementary Materials for

Color and near infrared reflectance covary in distinct ways across taxa

Jonathan Goldenberg, Jessica L. Dobson, Gerben Debruyn, Michaël P.J. Nicolaï, Svana Rogalla, Bram Vanthournout, Federico Massetti, Susana Clusella-Trullas, Diederik Strubbe, Katrien De Wolf , Dries Bonte, Bastiaan Star, Matthew D. Shawkey, Liliana D'Alba

Corresponding authors: jonathan.goldenberg@ugent.be | liliana.dalbaaltamirano@naturalis.nl

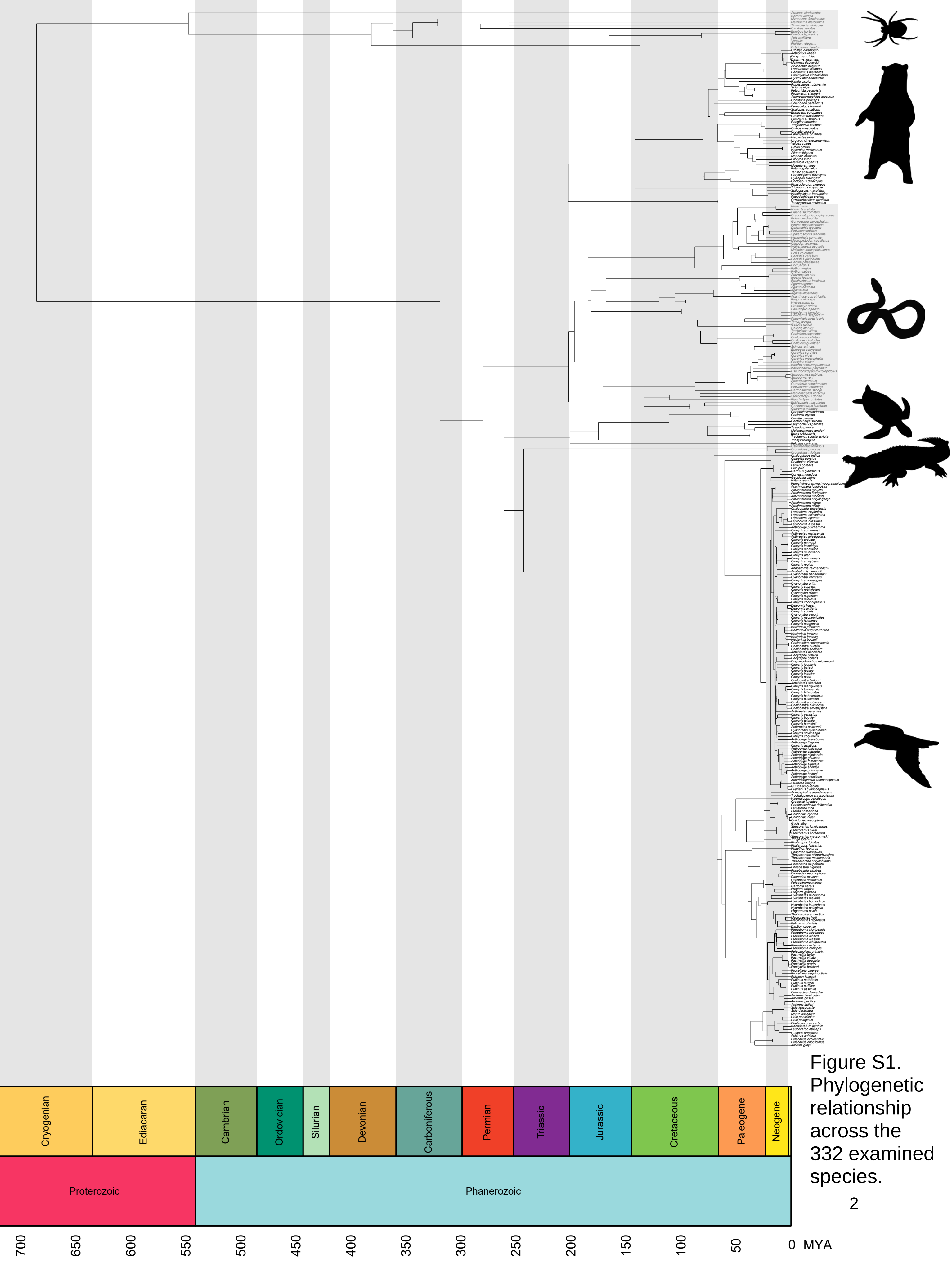

Figure S1. Phylogenetic relationship across the 332 examined species.

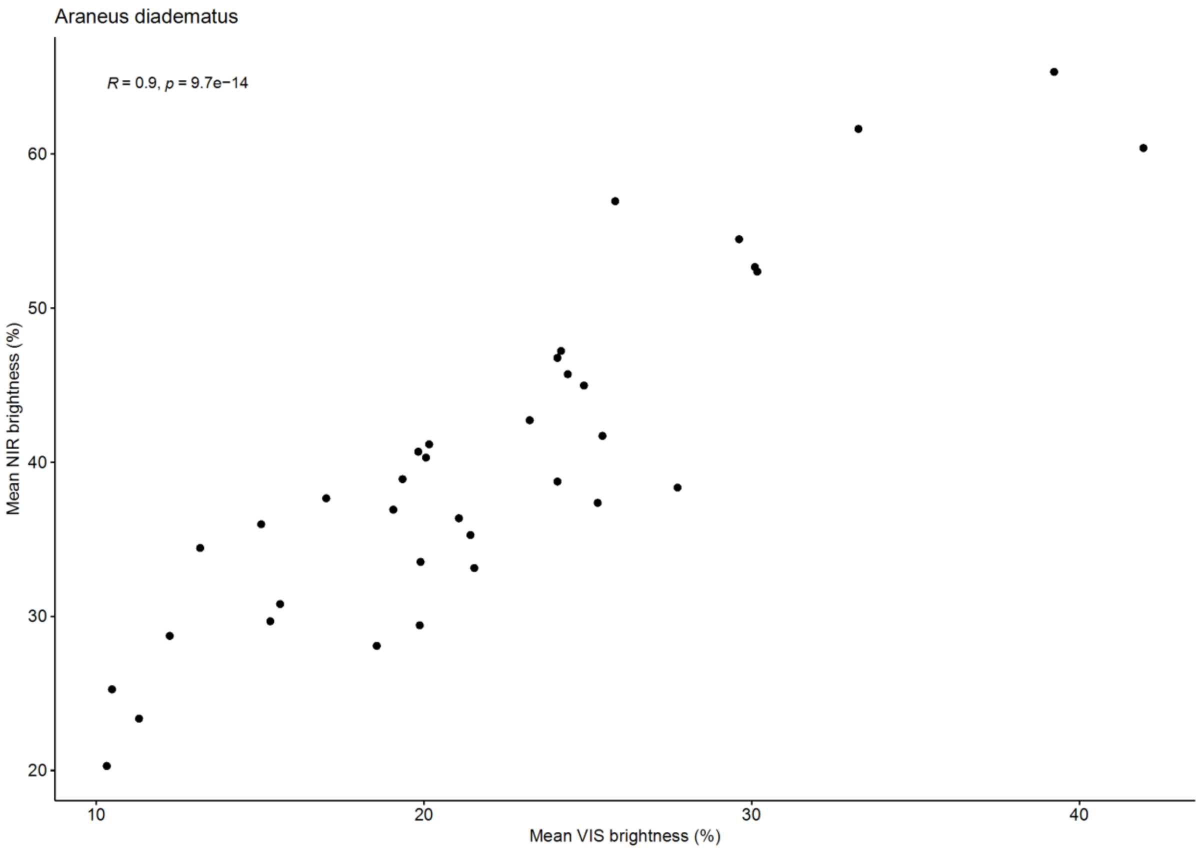

Figure S2. Variation in the relationship between Vis and NIR in individuals of the cross spider, *Araneus diadematus*, showing a strong positive correlation between the two spectral regions.

Table S1. Output from the phylogenetic regression model testing the relationship between UV and Vis spectra in integuments across the four examined animal groups. "ClassArthropoda" and "Vis:classArthropoda" are the two reference levels.

	Estimate	Std. Error	t value	Pr(>|t|)	
(Intercept)	-1.88985	8.16821	-0.2314	0.81721	
Vis	0.83543	0.19715	4.2376	3.12E-05	***
classAves	5.71651	12.80919	0.4463	0.65576	
classMammalia	7.91259	12.32696	0.6419	0.5215	
classSquamata	-4.0813	12.27312	-0.3325	0.73974	
Vis:classAves	-0.41139	0.20038	-2.053	0.04105	*
Vis:classMammalia	-0.41987	0.23802	-1.764	0.07887	.
Vis:classSquamata	-0.1009	0.21049	-0.4794	0.63207	

Signif. codes: 0 '***' 0.001 '**' 0.01 '*' 0.05 '.' 0.1 ' ' 1

Table S2. Output from the phylogenetic regression model testing the relationship between UV and Vis spectra in eggshells across the four examined animal groups. "ClassAves" and "Vis:classAves" are the two reference levels.

	Estimate	Std. Error	t value	Pr(>|t|)	
(Intercept)	-9.67754	7.150566	-1.3534	0.181368	
Vis.EGG	0.876437	0.122035	7.1818	1.73E-09	***
classCrocodylia	19.1682	40.76958	0.4702	0.640067	
classSquamata	5.477443	8.304887	0.6595	0.51225	
classTestudines	-38.6262	15.5243	-2.4881	0.015844	*
Vis.EGG:classCrocodylia	-0.04165	0.571529	-0.0729	0.94216	
Vis.EGG:classSquamata	0.061269	0.152332	0.4022	0.689061	
Vis.EGG:classTestudines	0.832107	0.238176	3.4937	0.000938	***

Signif. codes: 0 '***' 0.001 '**' 0.01 '*' 0.05 '.' 0.1 ' ' 1

Table S3. Output from the phylogenetic regression model testing the relationship between NIR and Vis spectra in integuments across the examined animal groups. "ClassArthropoda" and "Vis:classArthropoda" are the two reference levels.

	Estimate	Std. Error	t value	Pr(>|t|)	
(Intercept)	14.29215	9.579303	1.492	0.136884	
Vis	0.842412	0.245523	3.4311	0.000696	***
classAves	26.39115	15.62071	1.6895	0.092291	.
classMammalia	0.963626	15.0236	0.0641	0.948906	
classSquamata	-11.7105	14.95721	-0.7829	0.434361	
B2.VIS:classAves	-0.32247	0.249848	-1.2906	0.197944	
B2.VIS:classMammalia	0.5379	0.297689	1.8069	0.071901	.
B2.VIS:classSquamata	-0.04296	0.262566	-0.1636	0.870144	

Signif. codes: 0 '***' 0.001 '**' 0.01 '*' 0.05 '.' 0.1 ' ' 1

Table S4. Output from the posthoc test on the phylogenetic regression model testing the relationship between NIR and Vis spectra in integuments across the examined animal groups.

Contrast	Estimate	SE	z_value	p_value	
Aves vs Arthropoda	29.31611	7.555115	3.8803	1.04E-04	***
Mammalia vs Arthropoda	2.997061	7.555115	0.396693	0.691593	
Squamata vs Arthropoda	-9.09608	7.555115	-1.20396	0.228603	
Aves vs Mammalia	25.44724	7.555115	3.368213	7.57E-04	***
Aves vs Squamata	38.1215	7.555115	5.045787	4.52E-07	***
Mammalia vs Squamata	12.67426	7.555115	1.677574	0.093430	

Signif. codes: 0 '***' 0.001 '**' 0.01 '*' 0.05 '.' 0.1 ' ' 1

Table S5. Output from the phylogenetic regression model testing the relationship between NIR and Vis spectra in Aves.

	Estimate	Std. Error	t value	Pr(>|t|)	
(Intercept)	40.74773	2.540697	16.038	< 2.2e-16	***
Vis	0.501851	0.043335	11.581	< 2.2e-16	***

Signif. codes: 0 ‘***’ 0.001 ‘**’ 0.01 ‘*’ 0.05 ‘.’ 0.1 ‘ ’ 1

Table S6. Output from the phylogenetic regression model testing the relationship between Vis and NIR spectra in Mammalia.

	Estimate	Std. Error	t value	Pr(>|t|)	
(Intercept)	13.1351	1.6615	7.9058	3.06E-10	***
Vis	1.5114	0.1917	7.8839	3.30E-10	***

Signif. codes: 0 ‘***’ 0.001 ‘**’ 0.01 ‘*’ 0.05 ‘.’ 0.1 ‘ ’ 1

Table S7. Output from the phylogenetic regression model testing the relationship between Vis and NIR spectra in Squamata.

	Estimate	Std. Error	t value	Pr(>|t|)	
(Intercept)	2.51395	3.93068	0.6396	0.5259	
Vis	0.81536	0.10839	7.5227	2.62E-09	***

Signif. codes: 0 ‘***’ 0.001 ‘**’ 0.01 ‘*’ 0.05 ‘.’ 0.1 ‘ ’ 1

Table S8. Results from pairwise comparisons using Fisher's Z-test to assess statistical differences in correlation strength (R) across taxa in the phylogenetic regression model testing variability in NIR-Vis spectra in integuments (Tab. S3). Upper table shows the R values and the bottom one the pairwise comparisons.

Group	R
Aves	0.743
Squamata	0.816
Mammalia	0.751
Arthropoda	0.878

Comparison	p_value
Aves vs Arthropoda	0.230131
Aves vs Squamates	0.302041
Aves vs Mammals	0.927267
Arthropoda vs Squamata	0.530808
Arthropoda vs Mammalia	0.276893
Squamata vs Mammalia	0.440303

Signif. codes: 0 '***' 0.001 '**' 0.01 '*' 0.05 '.' 0.1 ' ' 1

Table S9. Output from the phylogenetic regression model testing the relationship between NIR and Vis spectra in eggshells across the four examined animal groups. "ClassAves" and "Vis:classAves" are the two reference levels.

	Estimate	Std. Error	t value	Pr(>|t|)	
(Intercept)	86.67375	5.076911	17.0721	< 2.2e-16	***
Vis.EGG	-0.07905	0.086645	-0.9123	0.36551	
classCrocodylia	-119.016	28.94646	-4.1116	0.00013	***
classSquamata	-64.1126	5.896481	-10.873	2.00E-15	***
classTestudines	-72.785	11.02227	-6.6034	1.56E-08	***
Vis.EGG:classCrocodylia	1.51593	0.405787	3.7358	0.000441	***
Vis.EGG:classSquamata	0.839719	0.108156	7.764	1.89E-10	***
Vis.EGG:classTestudines	0.930997	0.169105	5.5054	9.55E-07	***

Signif. codes: 0 '***' 0.001 '**' 0.01 '*' 0.05 '.' 0.1 ' ' 1

Table S10. Output from the posthoc test on the phylogenetic regression model testing the relationship between Vis and NIR spectra in eggshells across the four examined animal groups.

Contrast	Estimate	SE	z_value	p_value	
Crocodylia vs Aves	-120.532	7.699139	-15.6553	<< 0.01	***
Squamata vs Aves	-64.9523	7.699139	-8.43631	<< 0.01	***
Testudines vs Aves	-73.716	7.699139	-9.57458	<< 0.01	***
Crocodylia vs Squamata	-54.9035	7.699139	-7.13112	<< 0.01	***
Crocodylia vs Testudines	-46.2311	7.699139	-6.0047	<< 0.01	***
Squamata vs Testudines	8.672429	7.699139	1.126415	0.259	

Signif. codes: 0 ‘***’ 0.001 ‘**’ 0.01 ‘*’ 0.05 ‘.’ 0.1 ‘ ’ 1

Table S11. Results from pairwise comparisons using Fisher’s Z-test to assess statistical differences in correlation strength (R) across taxa in the phylogenetic regression model testing variability in NIR-Vis spectra in eggs (Tab. S9). Upper table shows the R values and the bottom one the pairwise comparisons.

Group	R
Aves	0.268
Crocodylia	0.989
Squamata	0.876
Testudines	0.957

Comparison	p_value
Aves vs Crocodylia	1
Aves vs Squamata	0.027046*
Aves vs Testudines	0.618424
Crocodylia vs Squamata	1
Crocodylia vs Testudines	1
Squamates vs Testudines	0.242012

Signif. codes: 0 ‘***’ 0.001 ‘**’ 0.01 ‘*’ 0.05 ‘.’ 0.1 ‘ ’ 1
